# Qualitative Simulation of Photon Transport in Free Space Based on Monte Carlo Method and Its Parallel Implementation

**DOI:** 10.1155/2010/650298

**Published:** 2010-06-27

**Authors:** Xueli Chen, Xinbo Gao, Xiaochao Qu, Duofang Chen, Bin Ma, Lin Wang, Kuan Peng, Jimin Liang, Jie Tian

**Affiliations:** ^1^Video and Image Processing System Lab, School of Electronic Engineering, Xidian University, Xi'an, Shaanxi 710071, China; ^2^Life Sciences Research Center, School of Life Sciences and Technology, Xidian University, Xi'an, Shaanxi 710071, China; ^3^Institute of Automation, Chinese Academy of Sciences, Beijing 100190, China

## Abstract

During the past decade, Monte Carlo method has obtained wide applications in optical imaging to simulate photon transport process inside tissues. However, this method has not been effectively extended to the simulation of free-space photon transport at present. In this paper, a uniform framework for noncontact optical imaging is proposed based on Monte Carlo method, which consists of the simulation of photon transport both in tissues and in free space. Specifically, the simplification theory of lens system is utilized to model the camera lens equipped in the optical imaging system, and Monte Carlo method is employed to describe the energy transformation from the tissue surface to the CCD camera. Also, the focusing effect of camera lens is considered to establish the relationship of corresponding points between tissue surface and CCD camera. Furthermore, a parallel version of the framework is realized, making the simulation much more convenient and effective. The feasibility of the uniform framework and the effectiveness of the parallel version are demonstrated with a cylindrical phantom based on real experimental results.

## 1. Introduction

As a gold standard of probabilistic statistics, Monte Carlo (MC) method has been widely applied in the simulation of various physical processes and used to achieve its statistical characteristic by sampling enormous random samples [1]. Among these applications, a stochastic model is firstly constructed, in which the expected value of a certain random variable or a random variable combination is equal to the physical quantity in the physical process; and then the expected value is determined by averaging the multi-sample of the random variable. Since MC simulation was first introduced to the field of laser-tissue interactions by Wilson and Adam [2], it has been applied widely in simulating photon transport in tissues and been improved a lot [3–7]. Furthermore, a software platform, a Monte Carlo model of steady-state light transport in multilayered tissues (MCML), was firstly developed by Wang's group to model the photon transport in multi-layered tissues [8]. Although the platform is portable to multiple computer platforms and several simulation results can be obtained, it is limited in normally incident light beam, simple geometries, and external source. To handle the aforementioned problems, Tian's group developed a new simulation platform, Molecular Optical Simulation Environment (MOSE), to simulate photon transport process in complicated and irregular geometries with internal light sources [9, 10]. However, MC method has not been effectively extended to simulate the photon transport process in free space. Theoretically, the simulation of photon transport in free space is an awkward problem, which is different from the simulation of photon transport in tissues. In free space, the complexity of the optical imaging system induces difficulties to the photon transport simulation. The presence of camera lens further complicates the photon transport process in free-space. Therefore, accurate and effective free-space photon transport model and the development of a uniform framework for the simulation of noncontact optical imaging are demanded urgently.

In this paper, a MC-based free-space photon transport (MC-FPT) model is developed, where the focusing effect of camera lens is taken into account and the simplification theory is used to model the camera lens as a thin lens. The mapping relationship of corresponding points between tissue surface and CCD camera is established using the focusing effect of the simplified thin lens. Furthermore, a uniform framework is constructed by incorporating the MC-FPT model into the MOSE platform, including the photon transport process from photon generation by light source to signal detection with a CCD camera. A parallel version of the uniform framework is realized based on MPICH which is a robust and flexible implementation and application of the message passing interface. Experiment using cylindrical phantom demonstrates the performance of the proposed model and further shows the potential of the parallel uniform framework for practical noncontact optical imaging applications.

## 2. Methods

### 2.1. Monte Carlo Method

MC method is a probability and statistical theory-based computation method, which achieves the statistical characteristic of physical process by sampling enormous random samples. It relies on random sampling of variables from well-defined probability distributions to compute the value of physical quantities. Using the MC method, a random statistical model should be constructed first, in which several random variables are defined and each corresponds to a physical quantity with predefined probability distribution, and then enough samples of the random variables are carried to achieve the simulation of the physical process reliably. Given a random variable *u* with probability density function *p*(*u*) defined in the interval (*a*, *b*), arbitrary sample *x* of the random variable will produce a random number *ξ* and satisfies

(1) $$\overset{x}{\underset{a}{\int }}p\left(u\right)du=\xi ,$$

where *x* is located in the interval (*a*, *b*) and *ξ* is in (0,1). Making use of (1), we can first generate the random number *ξ* by a pseudorandom number generator, and then sample the random *u* by solving (1) for *x*.

### 2.2. Photon Transport in Tissues

Photon transport in tissues is a complicated process and involves several activities including photon absorption, scattering, internal reflection, and transmission. To simulate photon transport process in tissues using MC method, the problems such as photon generation, photon movement, boundary effect, photon absorption, photon scattering, and photon termination should be concerned [9]. In practical applications, photon is commonly replaced by photon package. The power of each photon package is determined through dividing the total power of light source by the number of the photon package. Once a photon package is generated from the light source, it first moves a distance determined by the step size along its initial emitting direction, then it loses part of its power due to the absorption and change its movement direction, because of the scattering, before it moves to the next position. This process is repeated until the photon package is totally absorbed (marked as photon_dead = 1) or transmited through the outer boundary (marked as photon_dead = 0). In addition, the internal and the outer boundary effects should be concerned in the simulation.

During the photon transport process, the crucial step is to determine the next interaction site or the step size of the photon package, which can be calculated using the following equation:

(2) $$d=\frac{-\ln\;\zeta }{\mu_{a}+\mu_{s}},$$

where *d* is the step size of the photon package; *ζ* is a uniform random number in the interval (0,1); *μ*_*a*_ and  *μ*_s_ are the absorption and scattering coefficients, respectively.

For the statistical characteristics of MC method, more than 10^6^ photon packages are required to guarantee the accuracy and randomness of the simulation results. The more photon packages running in the simulation, the better the simulation results would be. Therefore, 10^8^ photon packages are used for all the simulations in this paper.

### 2.3. Photon Transport in Free Space

#### 2.3.1. Simplification of Optical Imaging System

Optical imaging system usually includes two inevitable parts, camera lens and CCD camera. The camera lens is utilized to transform and restrict the light beam, and the CCD camera is used to register the light beam that is selected by the camera lens. Therefore, the simplification of optical imaging system is composed of the simplification of the camera lens and the modeling of the CCD camera. The following section will present the simplification of the optical imaging system employed in the proposed MC-FPT model.

**Figure 1:**
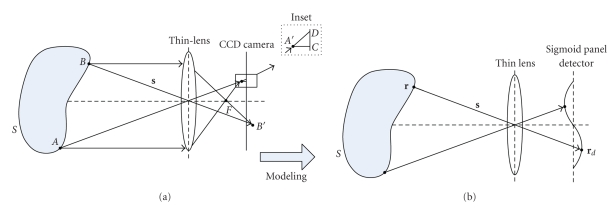
Schematic diagram for the simplification of optical imaging system.

A camera lens in an ideal optical system is typically not a single-lens, but a combination of a lens system with finite thickness and several other physical counterparts, such as camera lens diaphragms. Thus, photon transport in such combination is extremely complicated and difficult to analyze. Gauss had postulated that this combination can be simply modeled as a Gaussian thick lens by replacing it with two equivalent refracting surfaces [11, 12]. Furthermore, Gaussian thick lens can be equivalently reduced to a thin lens model based on geometric and photometric mapping [12]. Because the thin lens model exhibits several excellent features such as simplified structure and rectilinear propagation of light beam in the thin lens, the camera lens in the optical imaging system is approximate to the simplified thin-lens model, shown in Figure 1. Thus, the thin-lens-based lens law given by 1/*u* + 1/*v* = 1/*f* can be employed to establish the corresponding relationship between the object and image point as follows:

(3) $$r_{d}=r+\frac{u+v}{\cos\;\;\alpha }s,$$

where **r** is the coordinates of the object point at the tissue surface; **r**_*d*_ is the coordinates of the corresponding image point; *u* is the distance between the object point and the thin lens; *v* is the corresponding image distance determined by the lens law of the thin lens; *f* is the focal length of the thin lens; **s** represents the direction of the light beam that emits from tissue surface and passes though the axis center of the thin lens; cos  *α* is the cosine value of the angle between the direction **s** and optical axis.

For the irregularity of tissue surface, not all the object points at the tissue surface are exactly imaged at the CCD camera, as shown in Figure 1(a). Considering the focusing effect of the thin lens, this contribution assumes that all the surface points are in the range of depth-of-field. Each point at the tissue surface corresponds to one point at the CCD camera. As a result, image points, not at the CCD camera should be projected onto the camera. Two projection methods, including orthographic and perspective projection method, can be used to perform the projection process, as described in the inset of Figure 1(a). The trail A′C shows the orthographic projection process, and the trail A′D presents the perspective projection. Because Schulz had demonstrated that the perspective projection method could better improve image aberrations compared with the orthographic projection method [13], the perspective projection method is employed in the proposed MC-FPT model. On the basis of the aforementioned analysis, the CCD camera in the optical imaging system is modeled as a sigmoid panel detector to enhance the focusing effect-based, one-for-one mapping, as shown in Figure 1(b). Thus, an image point **r**_*d*_ on the sigmoid panel detector which might be the image point or the image facula center of the surface point **r** can be calculated by rewriting (3) as follows:

(4) $$r_{d}=r+\frac{u+v^{\prime }}{\cos\;\;\alpha }s,$$

where *v*′ is the distance from the image point **r**_*d*_ to the thin lens.

#### 2.3.2. Monte Carlo- Based Free-Space Photon Transport Model

Once the photon package reaches the outer boundary and no total reflection occurs, it would continue propagating in free space and the free-space photon transport model can be constructed using MC method. For this purpose, an assumption should be made first, that is, the free space is defined as a specific tissue which is assigned specific optical properties: the absorption coefficient *μ*_*a*_ = 0, the scattering coefficient *μ*_*s*_ = 0, and the anisotropy factor *g* = 1. According to (2), we obtain the step size of the photon package by the following formula:

(5) $$d=\frac{-\ln\;\zeta }{\mu_{a}+\mu_{s}}=\infty .$$

Equation (5) implies that the step size of the photon package approaches infinity when the photon package propagates in free space. Because the free space is assumed as a totally forward scatter, we can conclude that the photon package would continue propagating in its emitting direction until it hits an obstruction or is collected by the CCD camera. In the subsequent section, two mapping relationships between the tissue surface and the CCD camera, including spatial position and photon flux, are constructed to describe the implementation of the proposed MC-FPT model.

**(a) Mapping Relationship of Spatial Position between Tissue Surface and CCD Camera**

**Figure 2:**
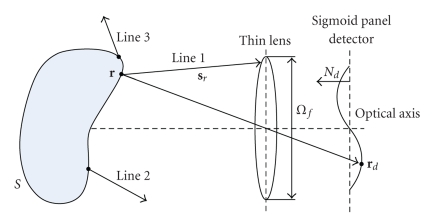
Schematic diagram for non-contact experimental setup.

Based on the aforementioned analysis, optical imaging system used in the imaging experiment can be equivalently modeled as a simple system, a combination of a simplified thin lens and a sigmoid panel detector. The experimental setup for this simplified non-contact measurement in real experiments is shown in Figure 2. Here, a visibility factor is introduced to be used as a criterion for determining whether the mapping relationship of spatial position between the surface point and the corresponding image point is established. In mathematics, the visibility factor is defined as

(6) $$\varepsilon \left(r,r_{d}\right)=\alpha \left(r,r_{d}\right)\beta \left(r,\Omega_{f}\right),$$

where *ε*(**r**, **r**_*d*_) is the visibility factor that consists of two parts, field visibility factor and system visibility factor; *α*(**r**, **r**_*d*_) is the field visibility factor that discards the outgoing photon packages not imaged at the detection point **r**_*d*_ and can be determined by

(7) $$\alpha \left(r,r_{d}\right)=\{\begin{array}{cc} 1, & \text{If}\;\;r_{d}=r+\frac{u+v^{\prime }}{\cos\;\;\alpha \;}\text{s},\\ 0, & \text{otherwise}. \end{array}$$

The system visibility factor is used to discard the outgoing photon packages that cannot reach the thin lens and defined as

(8) $$\beta \left(r,\Omega_{f}\right)=\{\begin{array}{cc} 1, & \text{If}\;{\;s}_{r}\cap \Omega_{f}\neq \emptyset ,\\ 0, & \text{If}{\;\;s}_{r}\cap \Omega_{f}=\emptyset , \end{array}$$

where *β*(**r**, **Ω**_*f*_) is the system visibility factor; **Ω**_*f*_ is a thin-lens space with the same diameter as the lens system and can be obtained in the imaging experiment. The expression **s**_**r**_ ∩ **Ω**_*f*_ ≠ *∅* can be interpreted that the outgoing photon packages can pass through the thin lens, as Line 1 shown in Figure 2; similarly, **s**_**r**_∩**Ω**_*f*_ = *∅* shows that the intersection point between the line and thin lens space is out of the range of the thin lens space, as Line 2 and Line 3 shown in Figure 2.

Based on the aforementioned analysis, the mapping relationship of spatial position between the surface points and the corresponding image points can be established using (4) and (6).

**(b) Mapping Relationship of Photon Flux between Tissue Surface and CCD Camera**

Because the optical imaging system is reduced to a simple combination of a simplified thin lens and a sigmoid panel detector, the focusing effect is utilized to establish the relationship between the corresponding points at the tissue surface and the sigmoid panel detector. Incorporating the aforementioned analysis of MC method, we can obtain the following formula:

(9) $$P\left(r_{d}\right)=\int_{\text{S}}\tau \left(r,r_{d};f\right)\varepsilon \left(r,r_{d}\right)P\left(r\right)dS,$$

where **r** is the position of outgoing photon at the tissue surface; **r**_*d*_ is the corresponding image point or image facula center of the outgoing photon package at the sigmoid panel detector, which can be determined by (4); *P*(**r**) is the power of the outgoing photon package emitted from the outer boundary; *P*(**r**_*d*_) is the power detected at point **r**_*d*_; *τ*(**r**, **r**_*d*_; *f*) is the energy-loss coefficient of the optical imaging system, which is dependent upon multifactors and can be measured in the experiment, including the transmittance of the optical system and the position of and the distance between the point **r** and **r**_*d*_,; *ε*(**r**, **r**_*d*_) is the visibility factor and can be determined by (6).

To sum up, the flux distribution at the CCD camera can be calculated directly by solving the formula shown in (9). The uniform implementation framework for the proposed MC-FPT model is presented in Algorithm 1.

### 2.4. Parallel Implementation

In order to improve the simulation efficiency of the proposed uniform framework, a parallel version is developed based on a small-scale parallel system, which is realized on Windows operating system. The parallel system is constructed by several personal computers, and all the computers are connected to a 16- port Ethernet Switch with the data transfer rate of 100 megabits per second. In the simulation, one of the CPUs is selected as master processor and the others are served as slaver processors. The master processor is responsible for controlling the parallel program and simultaneously participating in computation, and the slavers are utilized for computation. In addition, message passing mechanism is employed for the communications among the processors in the parallel framework, which is concretely implemented on MPICH, a robust and flexible implementation and application of the message passing interface (MPI). 

**Figure 3:**
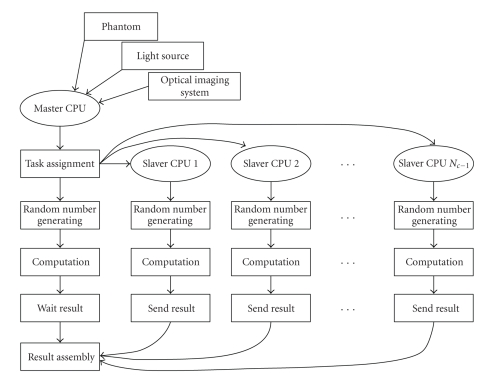
Flowchart of the proposed parallel uniform framework.

Pseudorandom generator is an important part of MC simulation, and the parallelization of the MC-based framework is to actually parallelize the pseudo-random generator. Because the master-slave mode communication is utilized in the proposed parallel framework, pseudo-random generator parallelization is realized by dividing cycle method [10]. Dividing cycle method is a common parallelization method to parallelize the pseudo-random generator and its implementation in the proposed parallel framework is described as follows. Firstly, a random number sequence is generated by the master processor and divided into several subdomains. Secondly, the first random number of each sub-domain is sent to the corresponding slaver processor as a seed. Finally, with the random number seeds, the slaver processors generate their own random number sequence. The whole flowchart of the proposed parallel framework is shown in Figure 3, where the task assignment includes three steps: first, a random number sequence is generated by the master CPU; second, the random number sequence is divided into *N*_*c*_ sub-domains, and *N*_*c*_ is the number of CPUs; third, the first random number of each sub-domain is sent to the corresponding slaver CPU. In addition, it should be noted that a multiplicative congruent generator is employed in the proposed MC-based framework, and the parallel program is coded with the standard C++ language.

## 3. Experiments and Results

**Figure 4:**
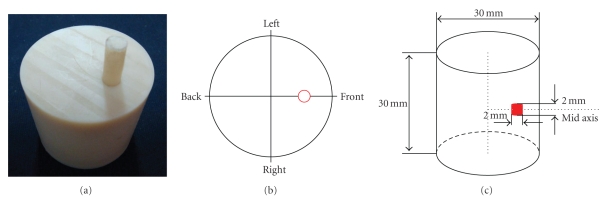
Experimental setup. (a) Physical phantom, (b) Four perspectives for CCD camera to register outgoing photons, (c) Numerical phantom.

A nylon cylindrical phantom of 30 mm diameter and 30 mm height was designed to verify the performance of the proposed MC-based free-space photon transport model and its parallel uniform framework. A small hole of 2 mm diameter and 16 mm depth from the top surface of the phantom was drilled with a distance of 8 mm from the center to emplace light source, as presented in literature [12]. The light source was made of luminescent solution that was extracted from a red luminescent light stick (Glow products, Canada). Because the central wavelength of luminescence light generated by the luminescent solution is about 650 nm, the optical properties of the phantom were measured at the wavelength about 660 nm by a time-correlated single photon counting (TCSPC) system [14]. The measured optical properties of the phantom are listed as follows: the absorption coefficient *μ*_*a*_ = 0.0138 mm^−1^ and the reduced scattering coefficient *μ*_*s*_′ = 0.91 mm^−1^. A cooled back-illuminated CCD camera (Princeton Instrument/Acton 2048B) coupled with a Nikkor Mrico 55 mm  f/2.8 camera lens was utilized to collect the emitted photons. In the experiment, the aperture value of the camera lens was set at f/8 to ensure that the front surface of the phantom was in the range of depth-of-field. The experimental setup is shown in Figure 4. Figure 4(a) shows the physical phantom used in the optical imaging experiment, Figure 4(b) describes four perspectives for the optical imaging system to register the outgoing luminescence light, and Figure 4(c) presents the numerical phantom utilized in the simulation framework.

### 3.1. Experiment Verifications

**Figure 5:**
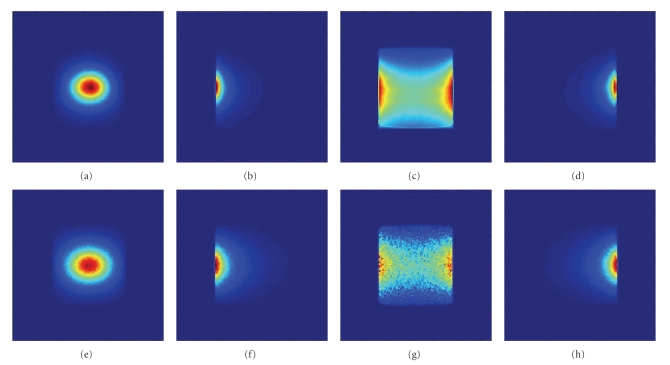
Flux distribution at the CCD camera. (a)–(d) Experimental data, (e)–(h) Simulation results, (a) and (e) front, (b) and (f) left, (c) and (g) back, (d) and (h) right perspective.

In this subsection, an optical imaging experiment is performed to validate the performance of the proposed MC framework using the aforementioned optical imaging system and experimental setup. Four perspective images; front, left, back, and right perspectives, are presented in Figure 5. Figures 5(a)–5(d) present the measured flux captured by the CCD camera in the imaging experiment, and Figures 5(e)–5(h) show the simulated flux of the proposed MC framework. Although similar tendency on flux distribution is achieved, there are still some flaws. Firstly, the simulated flux distribution is a little larger than the measured one, which is mainly caused by the simplification of optical imaging system and could be improved by more accurately modeling the optical imaging system. Secondly, the simulated flux is sparser than the measured one, which is intrinsically caused by the stochastic characteristic of MC method. Much smoother simulated flux can be obtained by increasing the number of photon package in the simulation.

**Figure 6:**
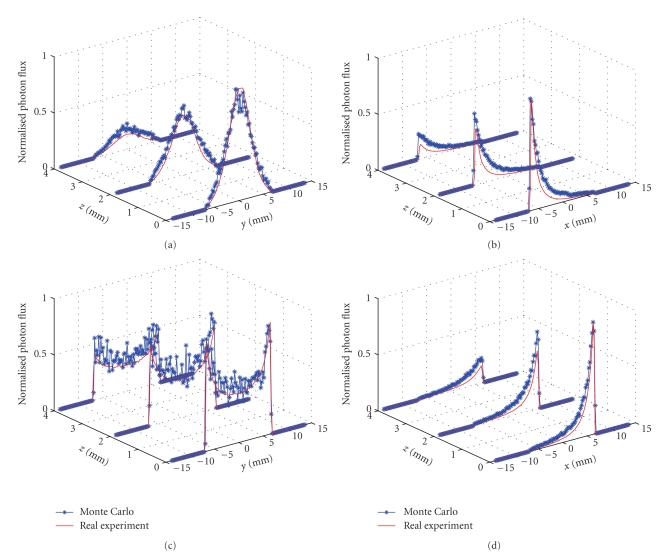
Comparison of MC simulation and real experiment at the detector position: *z*_*d*_ = 0.0, 2.0, 4.0 mm. (a)–(d) front-, left-, back- and right-perspective.

Results of detector *z* positions: *z*_*d*_ = 0.0,  2.0,  and 4.0 mm are examined and shown in Figure 6. Figures 6(a)–6(d) describe the comparison results between the proposed MC framework and real experiment. The solid lines show the results from real experiment, and the asterisks represent the simulated results of the proposed MC framework. Although the simulated flux seems a bit sparse, the mean error (ME) and root-mean-square error (RMSE) defined in literature [12] show the conformability from the aspect of quantitative error analysis, as listed in Table 1. Good agreement is observed in the cases examined, with the average ME and RMSE being about 0.0300 and 0.0015. Because the accuracy of the transmitted photon flux on the phantom surface simulated by MOSE has been sufficiently demonstrated [9, 10], the proposed MC framework works well in simulating photon transport in free space and provides great potential for non-contact optical imaging. 

### 3.2. Parallel Performance Demonstration

**Figure 7:**
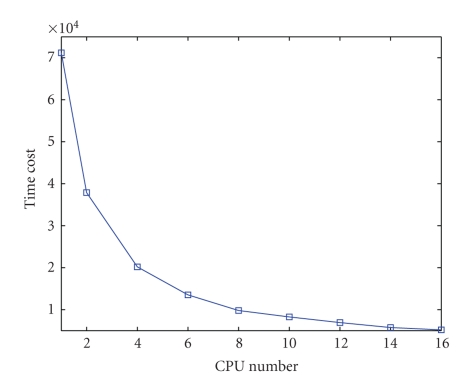
Performance comparison depending on CPU number.

Ideally, parallel execution on an increased number of CPUs should provide improved simulation performance. In order to evaluate the parallel uniform framework, performance demonstration experiment is performed using different number of CPUs. The experiment is performed on a workstation with two eight-core Xeon CPUs and 8 GB RAM. Figure 7 shows the variation of the simulation time with the CPU number. The total simulation time decreases with the increase of CPU number. Moreover, the downtrend of the simulation time tends to be steady when the number of CPUs increased to a certain value, 8 in this experiment. To further observe the improved performance on the simulation time, the factor of speedup ratio with respect to serial framework is calculated, as listed in Table 2. It also shows that the simulation time decreases to a relative stable value with the increase of CPU number. Since the simulation is performed on a group of CPUs, the communication time among the CPUs becomes significant with the increase of CPU number. As a result, the simulation time would less decrease when the number of CPUs reaches a certain value, as shown in Figure 7 and Table 2. Higher speed communication method can significantly reduce the simulation time cost. With the available hardware and software conditions, the number of CPUs must be predetermined to achieve a compromise between the simulation speed and the resources.

## 4. Discussions and Conclusion

In this paper, a statistical free space photon transport model is developed based on Monte Carlo method. It employs the simplification theory of the camera lens to simplify the optical imaging system and utilizes Monte Carlo method to describe the photon transport process in free space. Moreover, the focusing effect of the thin lens is introduced to depict the mapping relationship between the corresponding points at the tissue surface and CCD camera. Incorporating with photon transport simulation in tissues, we constructed a Monte-Carlo-based uniform framework for the forward problem of the non-contact optical imaging. Specially, a parallel version of the uniform framework was realized to improve the performance on speed. Comparisons with real experiment have demonstrated the feasibility and potential of the proposed uniform framework.

The proposed model can still be improved in the following aspects. Firstly, more accurate simplification model of optical imaging system can be constructed to improve the distribution of simulated photon flux, such as the consideration of the physical counterparts in the camera lens. Secondly, this paper just presents the qualitative study of photon transport in free space based on Monte Carlo method. Quantitative study can be realized by anther post processing method, such as the calibration of the CCD camera. Lastly, the proposed model is time consuming due to the statistical characteristic of Monte Carlo method. Although a parallel framework is realized to speedup the simulation, the parallel strategy is not perfect. Higher speed communication method or graphics processing unit (GPU) technique based on parallel strategy can be employed to better improve the simulation efficiency of the proposed model. Our future work will concentrate on the more accurate simplification model of the camera lens, the quantitative study of the proposed model, and the GPU technique based on acceleration. The corresponding results will be reported later.

## Figures and Tables

**Algorithm 1:**
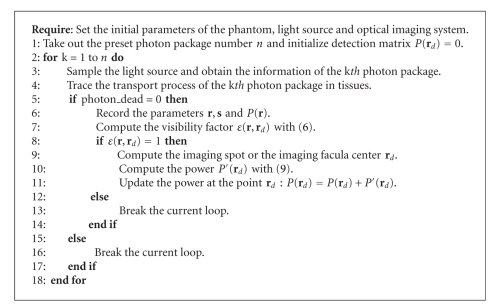
Uniform framework for MC-based photon transport model.

**Table 1:** Error comparisons between MC and real experiment.

	front	left	back	right	Average
ME	0.0253	0.0283	0.0417	0.0250	0.0300
RMSE	0.0013	0.0014	0.0021	0.0013	0.0015

**Table 2:** Performance comparison between parallel framework with different number of CPUs and serial framework. Speed-up Ratio is defined as the ratio between the time cost of multi-CPUs simulation and that of single CPU simulation. Relative error is defined as |Experiment value−Theoretical value|/ Theoretical value.

CPU number	Time Cost (*s*)	Speed-up Ratio		Relative error
		Experiment value	Theoretical value	
1	71173.4	1.00000	1.00000	0.00000
2	37898.9	1.87798	2.00000	0.06101
4	20200.9	3.52328	4.00000	0.11918
6	13526.8	5.26166	6.00000	0.12306
8	9802.32	7.26087	8.00000	0.09239
10	8271.94	8.60420	10.00000	0.13958
12	6926.80	10.2751	12.00000	0.14374
14	5758.62	12.3595	14.00000	0.11718
16	5199.02	13.6898	16.00000	0.14439
